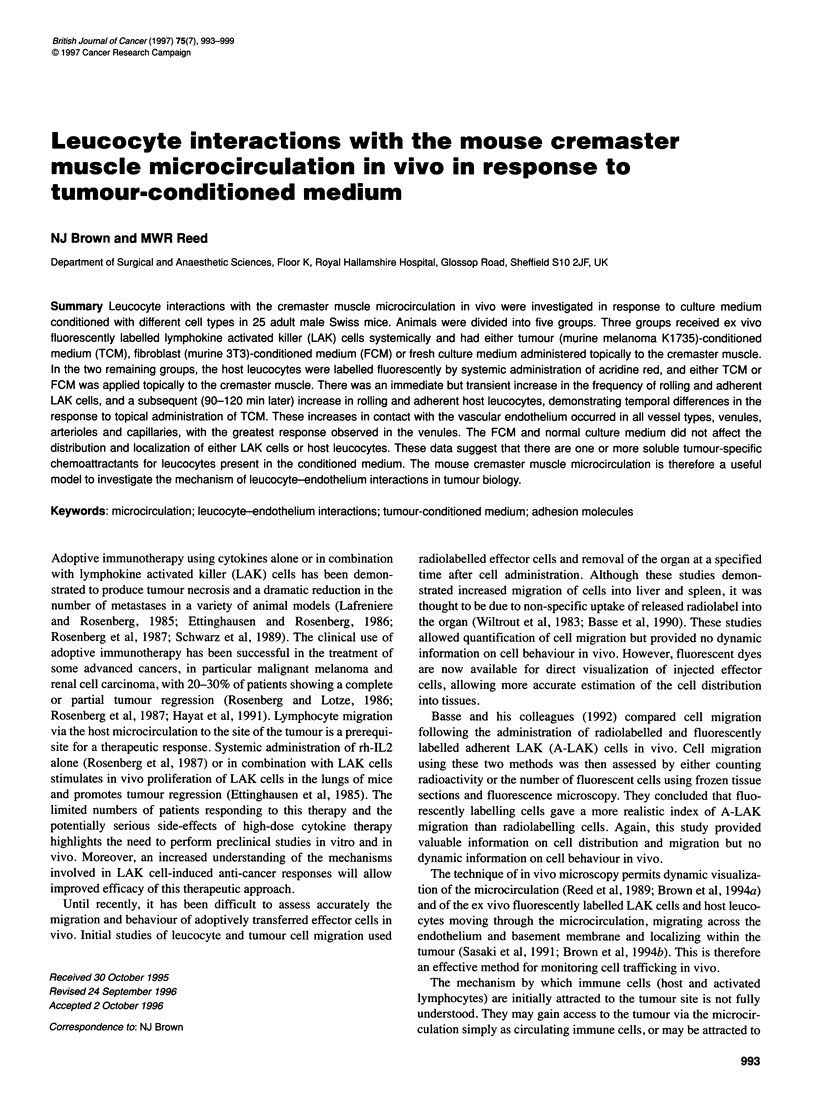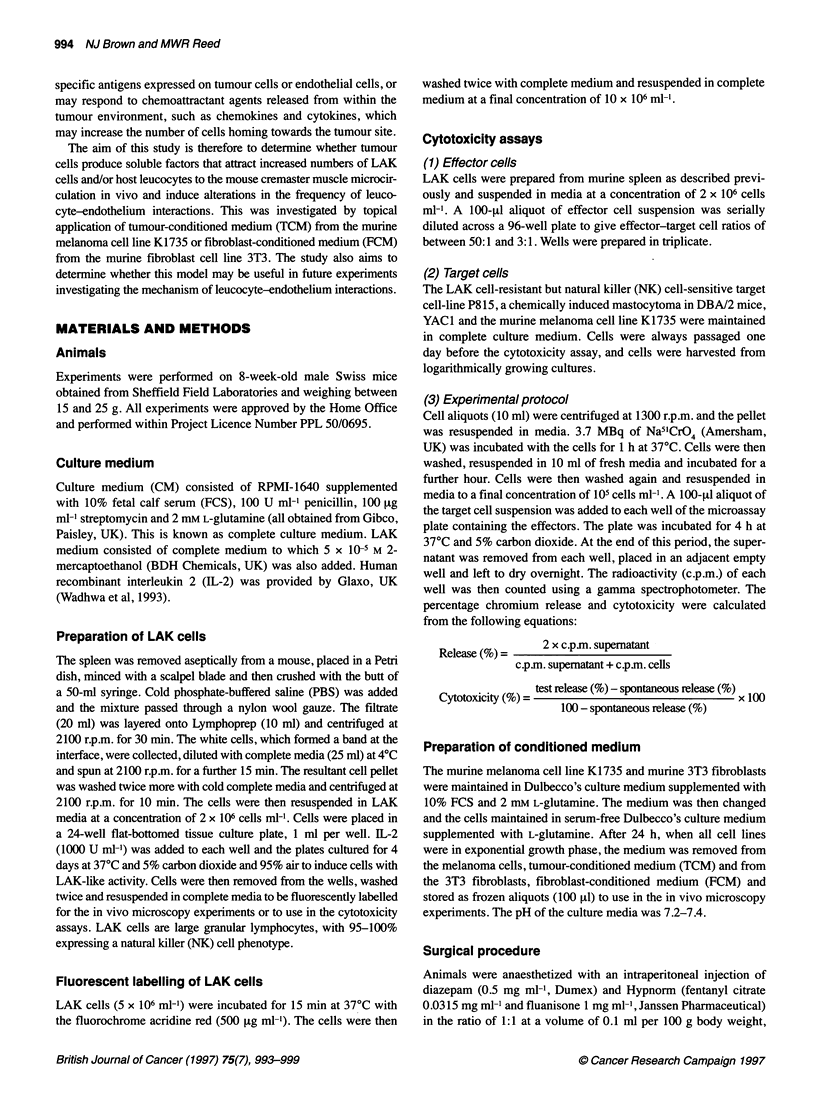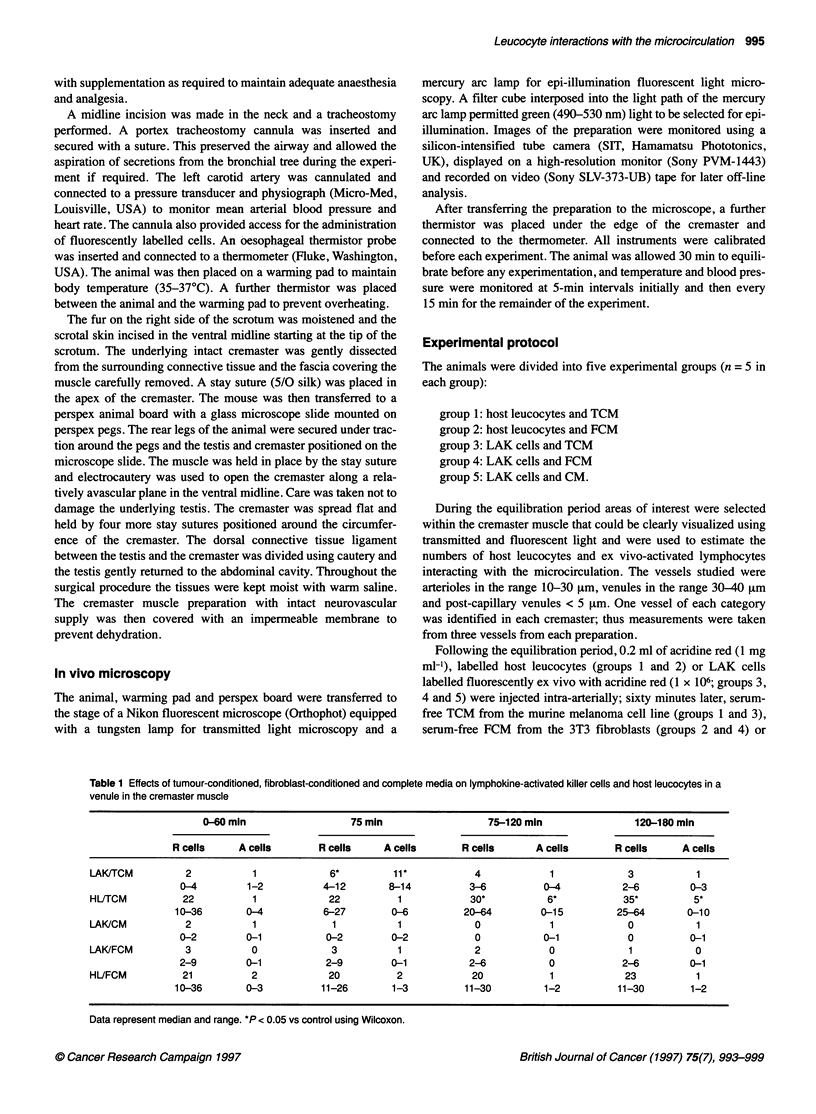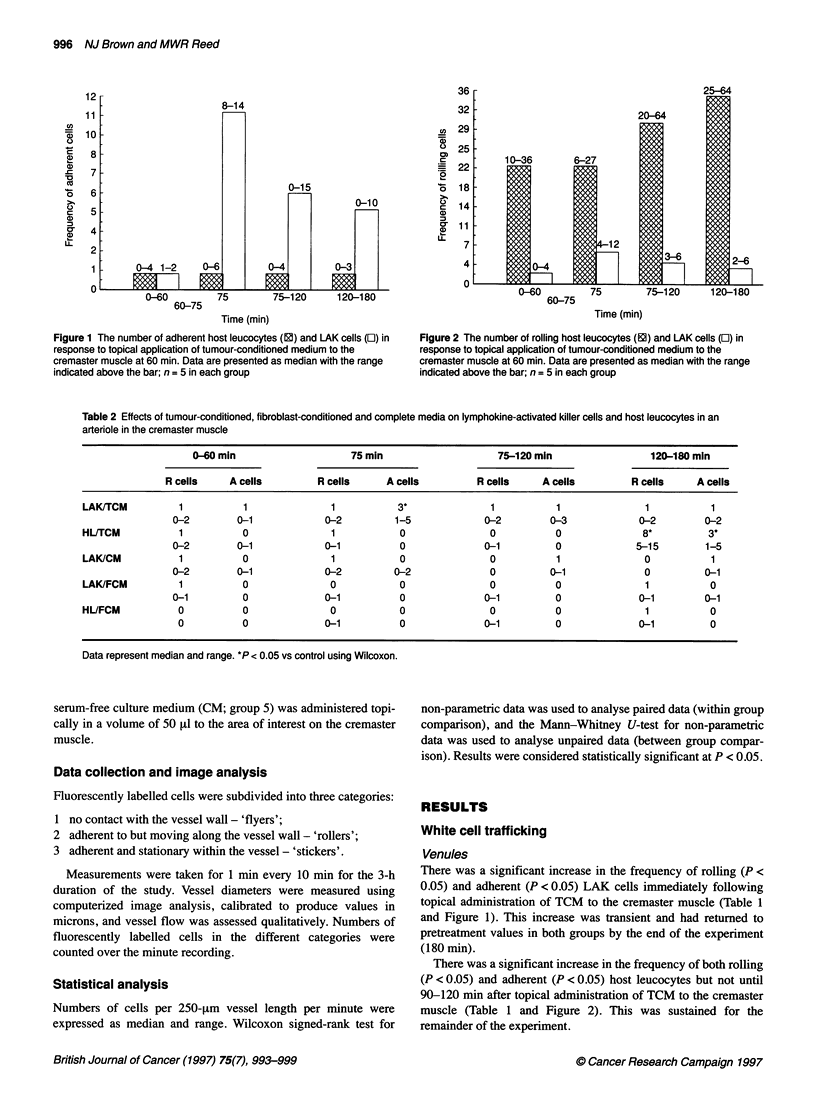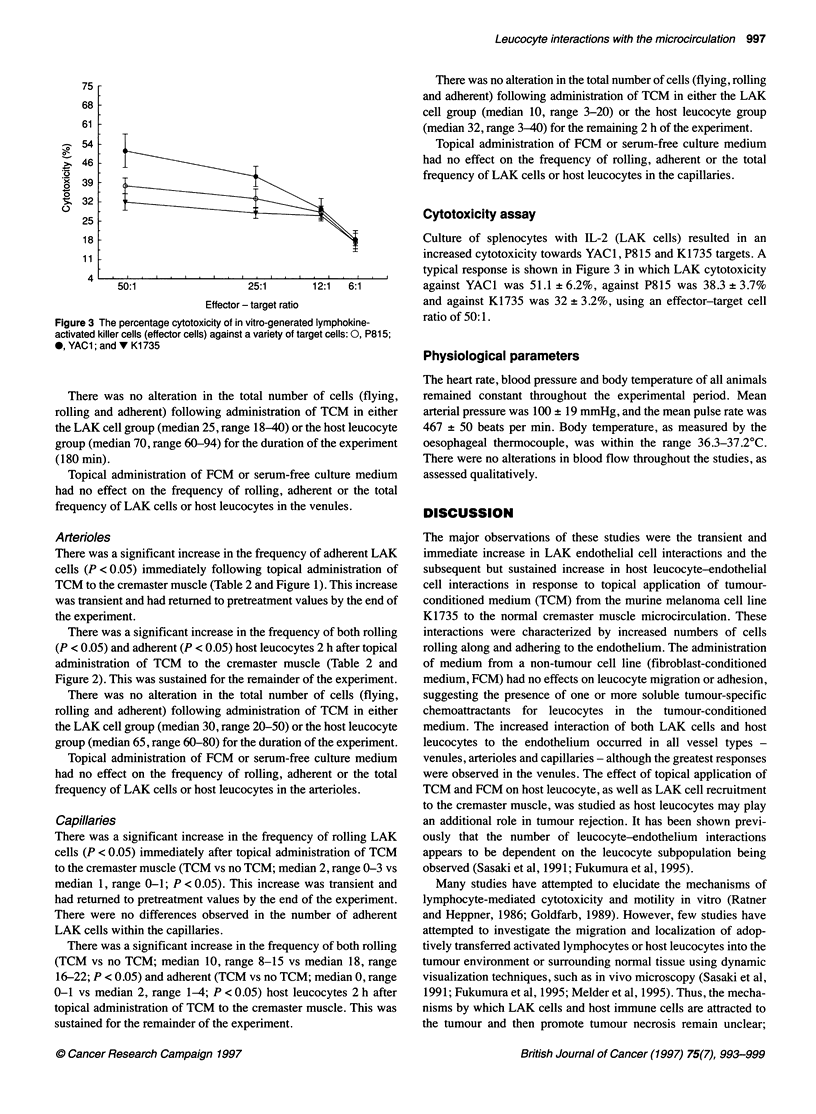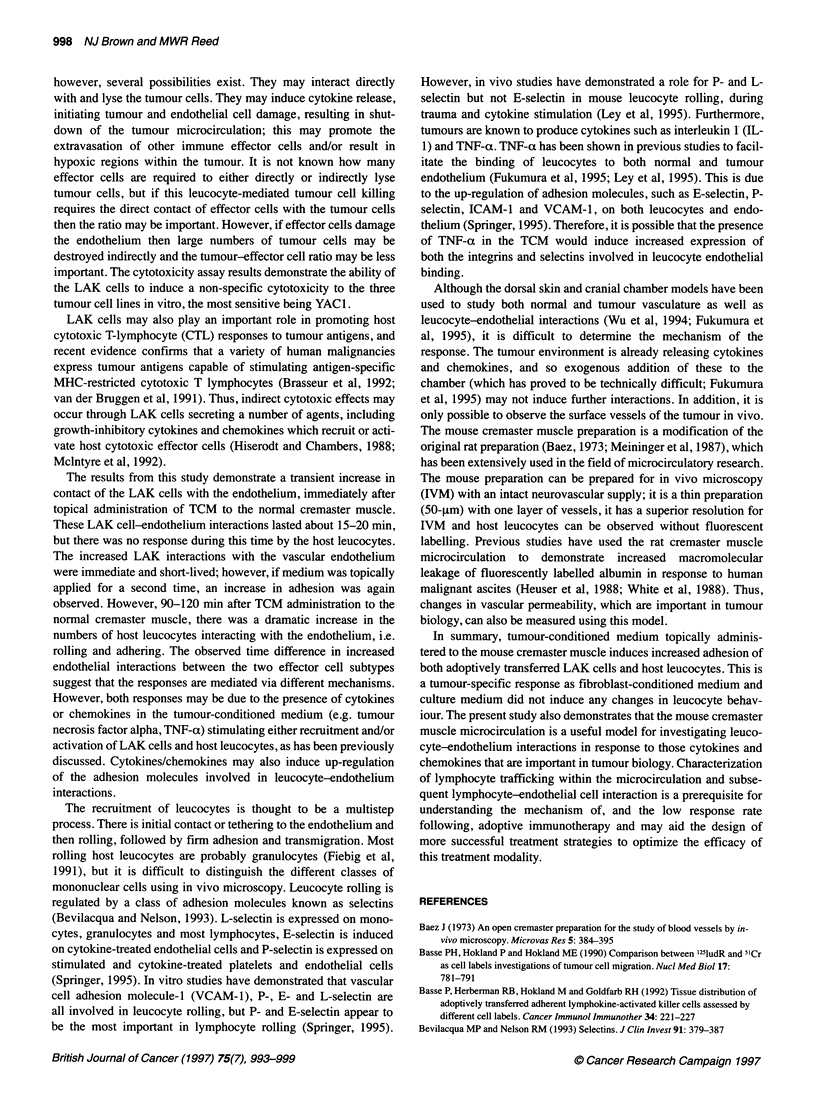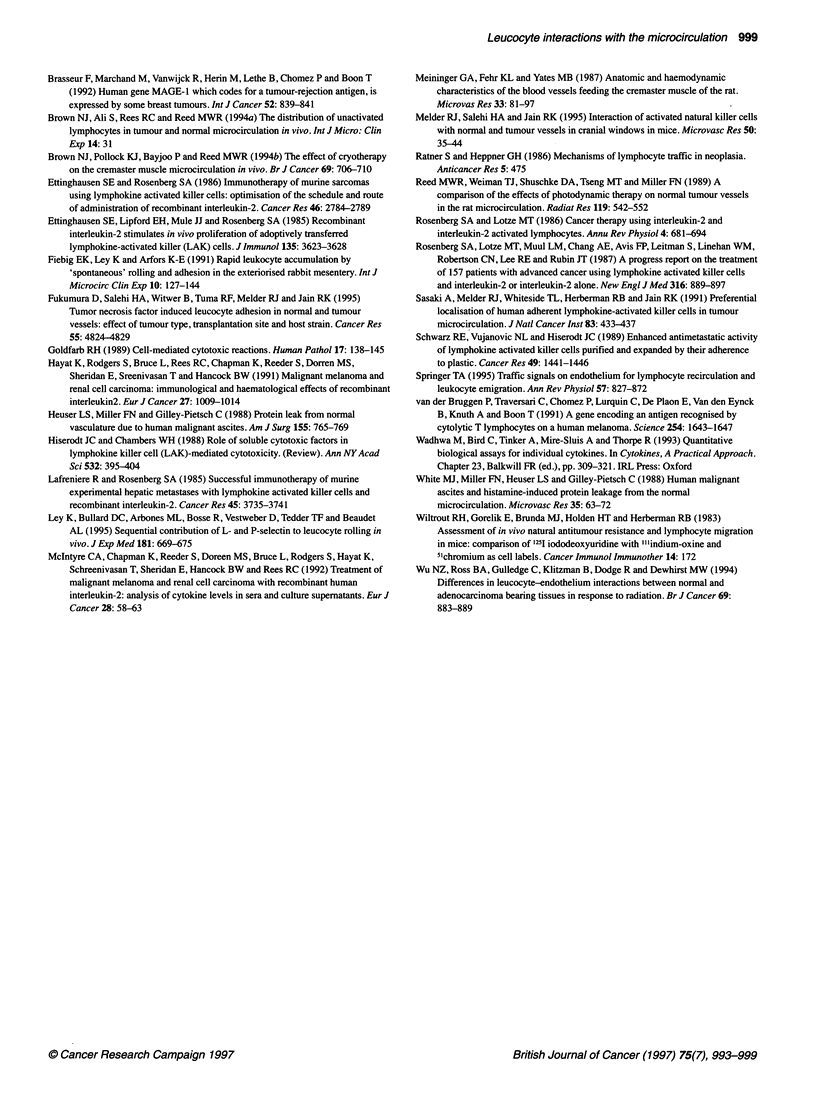# Leucocyte interactions with the mouse cremaster muscle microcirculation in vivo in response to tumour-conditioned medium.

**DOI:** 10.1038/bjc.1997.171

**Published:** 1997

**Authors:** N. J. Brown, M. W. Reed

**Affiliations:** Department of Surgical and Anaesthetic Sciences, Royal Hallamshire Hospital, Sheffield, UK.

## Abstract

Leucocyte interactions with the cremaster muscle microcirculation in vivo were investigated in response to culture medium conditioned with different cell types in 25 adult male Swiss mice. Animals were divided into five groups. Three groups received ex vivo fluorescently labelled lymphokine activated killer (LAK) cells systemically and had either tumour (murine melanoma K1735)-conditioned medium (TCM), fibroblast (murine 3T3)-conditioned medium (FCM) or fresh culture medium administered topically to the cremaster muscle. In the two remaining groups, the host leucocytes were labelled fluorescently by systemic administration of acridine red, and either TCM or FCM was applied topically to the cremaster muscle. There was an immediate but transient increase in the frequency of rolling and adherent LAK cells, and a subsequent (90-120 min later) increase in rolling and adherent host leucocytes, demonstrating temporal differences in the response to topical administration of TCM. These increases in contact with the vascular endothelium occurred in all vessel types, venules, arterioles and capillaries, with the greatest response observed in the venules. The FCM and normal culture medium did not affect the distribution and localization of either LAK cells or host leucocytes. These data suggest that there are one or more soluble tumour-specific chemoattractants for leucocytes present in the conditioned medium. The mouse cremaster muscle microcirculation is therefore a useful model to investigate the mechanism of leucocyte-endothelium interactions in tumour biology.


British Journal of Cancer (1997) 75(7), 993–999
© 1997 Cancer Research Campaign

### Leucocyte interactions with the mouse cremaster muscle microcirculation in vivo in response to tumour-conditioned medium

**NJ Brown and MWR Reed**

Department of Surgical and Anaesthetic Sciences, Floor K, Royal Hallamshire Hospital, Glossop Road, Sheffield S10 2JF, UK

**Summary** Leucocyte interactions with the cremaster muscle microcirculation in vivo were investigated in response to culture medium conditioned with different cell types in 25 adult male Swiss mice. Animals were divided into five groups. Three groups received ex vivo fluorescently labelled lymphokine activated killer (LAK) cells systemically and had either tumour (murine melanoma K1735)-conditioned medium (TCM), fibroblast (murine 3T3)-conditioned medium (FCM) or fresh culture medium administered topically to the cremaster muscle. In the two remaining groups, the host leucocytes were labelled fluorescently by systemic administration of acridine red, and either TCM or FCM was applied topically to the cremaster muscle. There was an immediate but transient increase in the frequency of rolling and adherent LAK cells, and a subsequent (90–120 min later) increase in rolling and adherent host leucocytes, demonstrating temporal differences in the response to topical administration of TCM. These increases in contact with the vascular endothelium occurred in all vessel types, venules, arterioles and capillaries, with the greatest response observed in the venules. The FCM and normal culture medium did not affect the distribution and localization of either LAK cells or host leucocytes. These data suggest that there are one or more soluble tumour-specific chemoattractants for leucocytes present in the conditioned medium. The mouse cremaster muscle microcirculation is therefore a useful model to investigate the mechanism of leucocyte–endothelium interactions in tumour biology.

**Keywords**: microcirculation; leucocyte–endothelium interactions; tumour-conditioned medium; adhesion molecules

Adoptive immunotherapy using cytokines alone or in combination with lymphokine activated killer (LAK) cells has been demonstrated to produce tumour necrosis and a dramatic reduction in the number of metastases in a variety of animal models (Lafreniere and Rosenberg, 1985; Ettinghausen and Rosenberg, 1986; Rosenberg et al, 1987; Schwarz et al, 1989). The clinical use of adoptive immunotherapy has been successful in the treatment of some advanced cancers, in particular malignant melanoma and renal cell carcinoma, with 20–30% of patients showing a complete or partial tumour regression (Rosenberg and Lotze, 1986; Rosenberg et al, 1987; Hayat et al, 1991). Lymphocyte migration via the host microcirculation to the site of the tumour is a prerequisite for a therapeutic response. Systemic administration of rh-IL2 alone (Rosenberg et al, 1987) or in combination with LAK cells stimulates in vivo proliferation of LAK cells in the lungs of mice and promotes tumour regression (Ettinghausen et al, 1985). The limited numbers of patients responding to this therapy and the potentially serious side-effects of high-dose cytokine therapy highlights the need to perform preclinical studies in vitro and in vivo. Moreover, an increased understanding of the mechanisms involved in LAK cell-induced anti-cancer responses will allow improved efficacy of this therapeutic approach.

Until recently, it has been difficult to assess accurately the migration and behaviour of adoptively transferred effector cells in vivo. Initial studies of leucocyte and tumour cell migration used radiolabelled effector cells and removal of the organ at a specified time after cell administration. Although these studies demonstrated increased migration of cells into liver and spleen, it was thought to be due to non-specific uptake of released radiolabel into the organ (Wiltrout et al, 1983; Basse et al, 1990). These studies allowed quantification of cell migration but provided no dynamic information on cell behaviour in vivo. However, fluorescent dyes are now available for direct visualization of injected effector cells, allowing more accurate estimation of the cell distribution into tissues.

Basse and his colleagues (1992) compared cell migration following the administration of radiolabelled and fluorescently labelled adherent LAK (A-LAK) cells in vivo. Cell migration using these two methods was then assessed by either counting radioactivity or the number of fluorescent cells using frozen tissue sections and fluorescence microscopy. They concluded that fluorescently labelling cells gave a more realistic index of A-LAK migration than radiolabelling cells. Again, this study provided valuable information on cell distribution and migration but no dynamic information on cell behaviour in vivo.

The technique of in vivo microscopy permits dynamic visualization of the microcirculation (Reed et al, 1989; Brown et al, 1994*a*) and of the ex vivo fluorescently labelled LAK cells and host leucocytes moving through the microcirculation, migrating across the endothelium and basement membrane and localizing within the tumour (Sasaki et al, 1991; Brown et al, 1994*b*). This is therefore an effective method for monitoring cell trafficking in vivo.

The mechanism by which immune cells (host and activated lymphocytes) are initially attracted to the tumour site is not fully understood. They may gain access to the tumour via the microcirculation simply as circulating immune cells, or may be attracted to

*Received 30 October 1995*
*Revised 24 September 1996*
*Accepted 2 October 1996*

*Correspondence to*: NJ Brown specific antigens expressed on tumour cells or endothelial cells, or may respond to chemoattractant agents released from within the tumour environment, such as chemokines and cytokines, which may increase the number of cells homing towards the tumour site.

The aim of this study is therefore to determine whether tumour cells produce soluble factors that attract increased numbers of LAK cells and/or host leucocytes to the mouse cremaster muscle microcirculation in vivo and induce alterations in the frequency of leucocyte–endothelium interactions. This was investigated by topical application of tumour-conditioned medium (TCM) from the murine melanoma cell line K1735 or fibroblast-conditioned medium (FCM) from the murine fibroblast cell line 3T3. The study also aims to determine whether this model may be useful in future experiments investigating the mechanism of leucocyte–endothelium interactions.

### MATERIALS AND METHODS

### Animals

Experiments were performed on 8-week-old male Swiss mice obtained from Sheffield Field Laboratories and weighing between 15 and 25 g. All experiments were approved by the Home Office and performed within Project Licence Number PPL 50/0695.

### Culture medium

Culture medium (CM) consisted of RPMI-1640 supplemented with 10% fetal calf serum (FCS), 100 U $ml^{-1}$ penicillin, 100 μg $ml^{-1}$ streptomycin and 2 mM L-glutamine (all obtained from Gibco, Paisley, UK). This is known as complete culture medium. LAK medium consisted of complete medium to which $5 \times 10^{-5}$ M 2-mercaptoethanol (BDH Chemicals, UK) was also added. Human recombinant interleukin 2 (IL-2) was provided by Glaxo, UK (Wadhwa et al, 1993).

### Preparation of LAK cells

The spleen was removed aseptically from a mouse, placed in a Petri dish, minced with a scalpel blade and then crushed with the butt of a 50-ml syringe. Cold phosphate-buffered saline (PBS) was added and the mixture passed through a nylon wool gauze. The filtrate (20 ml) was layered onto Lymphoprep (10 ml) and centrifuged at 2100 r.p.m. for 30 min. The white cells, which formed a band at the interface, were collected, diluted with complete media (25 ml) at 4°C and spun at 2100 r.p.m. for a further 15 min. The resultant cell pellet was washed twice more with cold complete media and centrifuged at 2100 r.p.m. for 10 min. The cells were then resuspended in LAK media at a concentration of $2 \times 10^6$ cells $ml^{-1}$. Cells were placed in a 24-well flat-bottomed tissue culture plate, 1 ml per well. IL-2 (1000 U $ml^{-1}$) was added to each well and the plates cultured for 4 days at 37°C and 5% carbon dioxide and 95% air to induce cells with LAK-like activity. Cells were then removed from the wells, washed twice and resuspended in complete media to be fluorescently labelled for the in vivo microscopy experiments or to use in the cytotoxicity assays. LAK cells are large granular lymphocytes, with 95–100% expressing a natural killer (NK) cell phenotype.

### Fluorescent labelling of LAK cells

LAK cells ($5 \times 10^6$ $ml^{-1}$) were incubated for 15 min at 37°C with the fluorochrome acridine red (500 μg $ml^{-1}$). The cells were then washed twice with complete medium and resuspended in complete medium at a final concentration of $10 \times 10^6$ $ml^{-1}$.

### Cytotoxicity assays

*(1) Effector cells*

LAK cells were prepared from murine spleen as described previously and suspended in media at a concentration of $2 \times 10^6$ cells $ml^{-1}$. A 100-μl aliquot of effector cell suspension was serially diluted across a 96-well plate to give effector–target cell ratios of between 50:1 and 3:1. Wells were prepared in triplicate.

*(2) Target cells*

The LAK cell-resistant but natural killer (NK) cell-sensitive target cell-line P815, a chemically induced mastocytoma in DBA/2 mice, YAC1 and the murine melanoma cell line K1735 were maintained in complete culture medium. Cells were always passaged one day before the cytotoxicity assay, and cells were harvested from logarithmically growing cultures.

*(3) Experimental protocol*

Cell aliquots (10 ml) were centrifuged at 1300 r.p.m. and the pellet was resuspended in media. 3.7 MBq of $Na^{51}CrO_4$ (Amersham, UK) was incubated with the cells for 1 h at 37°C. Cells were then washed, resuspended in 10 ml of fresh media and incubated for a further hour. Cells were then washed again and resuspended in media to a final concentration of $10^5$ cells $ml^{-1}$. A 100-μl aliquot of the target cell suspension was added to each well of the microassay plate containing the effectors. The plate was incubated for 4 h at 37°C and 5% carbon dioxide. At the end of this period, the supernatant was removed from each well, placed in an adjacent empty well and left to dry overnight. The radioactivity (c.p.m.) of each well was then counted using a gamma spectrophotometer. The percentage chromium release and cytotoxicity were calculated from the following equations:

$$\text{Release (\%)} = \frac{2 \times \text{c.p.m. supernatant}}{\text{c.p.m. supernatant} + \text{c.p.m. cells}}$$

$$\text{Cytotoxicity (\%)} = \frac{\text{test release (\%)} - \text{spontaneous release (\%)}}{100 - \text{spontaneous release (\%)}} \times 100$$

### Preparation of conditioned medium

The murine melanoma cell line K1735 and murine 3T3 fibroblasts were maintained in Dulbecco's culture medium supplemented with 10% FCS and 2 mM L-glutamine. The medium was then changed and the cells maintained in serum-free Dulbecco's culture medium supplemented with L-glutamine. After 24 h, when all cell lines were in exponential growth phase, the medium was removed from the melanoma cells, tumour-conditioned medium (TCM) and from the 3T3 fibroblasts, fibroblast-conditioned medium (FCM) and stored as frozen aliquots (100 μl) to use in the in vivo microscopy experiments. The pH of the culture media was 7.2–7.4.

### Surgical procedure

Animals were anaesthetized with an intraperitoneal injection of diazepam (0.5 mg $ml^{-1}$, Dumex) and Hypnorm (fentanyl citrate 0.0315 mg $ml^{-1}$ and fluanisone 1 mg $ml^{-1}$, Janssen Pharmaceutical) in the ratio of 1:1 at a volume of 0.1 ml per 100 g body weight,

© Cancer Research Campaign 1997

with supplementation as required to maintain adequate anaesthesia and analgesia.

A midline incision was made in the neck and a tracheostomy performed. A portex tracheostomy cannula was inserted and secured with a suture. This preserved the airway and allowed the aspiration of secretions from the bronchial tree during the experiment if required. The left carotid artery was cannulated and connected to a pressure transducer and physiograph (Micro-Med, Louisville, USA) to monitor mean arterial blood pressure and heart rate. The cannula also provided access for the administration of fluorescently labelled cells. An oesophageal thermistor probe was inserted and connected to a thermometer (Fluke, Washington, USA). The animal was then placed on a warming pad to maintain body temperature (35–37°C). A further thermistor was placed between the animal and the warming pad to prevent overheating.

The fur on the right side of the scrotum was moistened and the scrotal skin incised in the ventral midline starting at the tip of the scrotum. The underlying intact cremaster was gently dissected from the surrounding connective tissue and the fascia covering the muscle carefully removed. A stay suture (5/O silk) was placed in the apex of the cremaster. The mouse was then transferred to a perspex animal board with a glass microscope slide mounted on perspex pegs. The rear legs of the animal were secured under traction around the pegs and the testis and cremaster positioned on the microscope slide. The muscle was held in place by the stay suture and electrocautery was used to open the cremaster along a relatively avascular plane in the ventral midline. Care was taken not to damage the underlying testis. The cremaster was spread flat and held by four more stay sutures positioned around the circumference of the cremaster. The dorsal connective tissue ligament between the testis and the cremaster was divided using cautery and the testis gently returned to the abdominal cavity. Throughout the surgical procedure the tissues were kept moist with warm saline. The cremaster muscle preparation with intact neurovascular supply was then covered with an impermeable membrane to prevent dehydration.

### In vivo microscopy

The animal, warming pad and perspex board were transferred to the stage of a Nikon fluorescent microscope (Orthophot) equipped with a tungsten lamp for transmitted light microscopy and a mercury arc lamp for epi-illumination fluorescent light microscopy. A filter cube interposed into the light path of the mercury arc lamp permitted green (490–530 nm) light to be selected for epi-illumination. Images of the preparation were monitored using a silicon-intensified tube camera (SIT, Hamamatsu Phototonics, UK), displayed on a high-resolution monitor (Sony PVM-1443) and recorded on video (Sony SLV-373-UB) tape for later off-line analysis.

After transferring the preparation to the microscope, a further thermistor was placed under the edge of the cremaster and connected to the thermometer. All instruments were calibrated before each experiment. The animal was allowed 30 min to equilibrate before any experimentation, and temperature and blood pressure were monitored at 5-min intervals initially and then every 15 min for the remainder of the experiment.

### Experimental protocol

The animals were divided into five experimental groups ($n = 5$ in each group):

group 1: host leucocytes and TCM
group 2: host leucocytes and FCM
group 3: LAK cells and TCM
group 4: LAK cells and FCM
group 5: LAK cells and CM.

During the equilibration period areas of interest were selected within the cremaster muscle that could be clearly visualized using transmitted and fluorescent light and were used to estimate the numbers of host leucocytes and ex vivo-activated lymphocytes interacting with the microcirculation. The vessels studied were arterioles in the range 10–30 μm, venules in the range 30–40 μm and post-capillary venules < 5 μm. One vessel of each category was identified in each cremaster; thus measurements were taken from three vessels from each preparation.

Following the equilibration period, 0.2 ml of acridine red (1 mg ml$^{-1}$), labelled host leucocytes (groups 1 and 2) or LAK cells labelled fluorescently ex vivo with acridine red ($1 \times 10^6$; groups 3, 4 and 5) were injected intra-arterially; sixty minutes later, serum-free TCM from the murine melanoma cell line (groups 1 and 3), serum-free FCM from the 3T3 fibroblasts (groups 2 and 4) or

**Table 1** Effects of tumour-conditioned, fibroblast-conditioned and complete media on lymphokine-activated killer cells and host leucocytes in a venule in the cremaster muscle

| | 0–60 min | | 75 min | | 75–120 min | | 120–180 min | |
|---|---|---|---|---|---|---|---|---|
| | R cells | A cells | R cells | A cells | R cells | A cells | R cells | A cells |
| LAK/TCM | 2 | 1 | 6* | 11* | 4 | 1 | 3 | 1 |
| | 0–4 | 1–2 | 4–12 | 8–14 | 3–6 | 0–4 | 2–6 | 0–3 |
| HL/TCM | 22 | 1 | 22 | 1 | 30* | 6* | 35* | 5* |
| | 10–36 | 0–4 | 6–27 | 0–6 | 20–64 | 0–15 | 25–64 | 0–10 |
| LAK/CM | 2 | 1 | 1 | 1 | 0 | 1 | 0 | 1 |
| | 0–2 | 0–1 | 0–2 | 0–2 | 0 | 0–1 | 0 | 0–1 |
| LAK/FCM | 3 | 0 | 3 | 1 | 2 | 0 | 1 | 0 |
| | 2–9 | 0–1 | 2–9 | 0–1 | 2–6 | 0 | 2–6 | 0–1 |
| HL/FCM | 21 | 2 | 20 | 2 | 20 | 1 | 23 | 1 |
| | 10–36 | 0–3 | 11–26 | 1–3 | 11–30 | 1–2 | 11–30 | 1–2 |

Data represent median and range. *$P < 0.05$ vs control using Wilcoxon.

**Figure 1** The number of adherent host leucocytes (⊠) and LAK cells (□) in response to topical application of tumour-conditioned medium to the cremaster muscle at 60 min. Data are presented as median with the range indicated above the bar; $n = 5$ in each group

**Figure 2** The number of rolling host leucocytes (⊠) and LAK cells (□) in response to topical application of tumour-conditioned medium to the cremaster muscle at 60 min. Data are presented as median with the range indicated above the bar; $n = 5$ in each group

**Table 2** Effects of tumour-conditioned, fibroblast-conditioned and complete media on lymphokine-activated killer cells and host leucocytes in an arteriole in the cremaster muscle

| | 0–60 min | | 75 min | | 75–120 min | | 120–180 min | |
|---|---|---|---|---|---|---|---|---|
| | R cells | A cells | R cells | A cells | R cells | A cells | R cells | A cells |
| LAK/TCM | 1 | 1 | 1 | 3* | 1 | 1 | 1 | 1 |
| | 0–2 | 0–1 | 0–2 | 1–5 | 0–2 | 0–3 | 0–2 | 0–2 |
| HL/TCM | 1 | 0 | 1 | 0 | 0 | 0 | 8* | 3* |
| | 0–2 | 0–1 | 0–1 | 0 | 0–1 | 0 | 5–15 | 1–5 |
| LAK/CM | 1 | 0 | 1 | 0 | 0 | 1 | 0 | 1 |
| | 0–2 | 0–1 | 0–2 | 0–2 | 0 | 0–1 | 0 | 0–1 |
| LAK/FCM | 1 | 0 | 0 | 0 | 0 | 0 | 1 | 0 |
| | 0–1 | 0 | 0–1 | 0 | 0–1 | 0 | 0–1 | 0–1 |
| HL/FCM | 0 | 0 | 0 | 0 | 0 | 0 | 1 | 0 |
| | 0 | 0 | 0–1 | 0 | 0–1 | 0 | 0–1 | 0 |

Data represent median and range. *$P < 0.05$ vs control using Wilcoxon.

serum-free culture medium (CM; group 5) was administered topically in a volume of 50 µl to the area of interest on the cremaster muscle.

### Data collection and image analysis

Fluorescently labelled cells were subdivided into three categories:

1 no contact with the vessel wall – 'flyers';
2 adherent to but moving along the vessel wall – 'rollers';
3 adherent and stationary within the vessel – 'stickers'.

Measurements were taken for 1 min every 10 min for the 3-h duration of the study. Vessel diameters were measured using computerized image analysis, calibrated to produce values in microns, and vessel flow was assessed qualitatively. Numbers of fluorescently labelled cells in the different categories were counted over the minute recording.

### Statistical analysis

Numbers of cells per 250-µm vessel length per minute were expressed as median and range. Wilcoxon signed-rank test for non-parametric data was used to analyse paired data (within group comparison), and the Mann–Whitney *U*-test for non-parametric data was used to analyse unpaired data (between group comparison). Results were considered statistically significant at $P < 0.05$.

### RESULTS

### White cell trafficking

### *Venules*

There was a significant increase in the frequency of rolling ($P <$ 0.05) and adherent ($P < 0.05$) LAK cells immediately following topical administration of TCM to the cremaster muscle (Table 1 and Figure 1). This increase was transient and had returned to pretreatment values in both groups by the end of the experiment (180 min).

There was a significant increase in the frequency of both rolling ($P < 0.05$) and adherent ($P < 0.05$) host leucocytes but not until 90–120 min after topical administration of TCM to the cremaster muscle (Table 1 and Figure 2). This was sustained for the remainder of the experiment.

© Cancer Research Campaign 1997

**Figure 3** The percentage cytotoxicity of in vitro-generated lymphokine-activated killer cells (effector cells) against a variety of target cells: ○, P815; ●, YAC1; and ▼ K1735

There was no alteration in the total number of cells (flying, rolling and adherent) following administration of TCM in either the LAK cell group (median 25, range 18–40) or the host leucocyte group (median 70, range 60–94) for the duration of the experiment (180 min).

Topical administration of FCM or serum-free culture medium had no effect on the frequency of rolling, adherent or the total frequency of LAK cells or host leucocytes in the venules.

### *Arterioles*

There was a significant increase in the frequency of adherent LAK cells ($P < 0.05$) immediately following topical administration of TCM to the cremaster muscle (Table 2 and Figure 1). This increase was transient and had returned to pretreatment values by the end of the experiment.

There was a significant increase in the frequency of both rolling ($P < 0.05$) and adherent ($P < 0.05$) host leucocytes 2 h after topical administration of TCM to the cremaster muscle (Table 2 and Figure 2). This was sustained for the remainder of the experiment.

There was no alteration in the total number of cells (flying, rolling and adherent) following administration of TCM in either the LAK cell group (median 30, range 20–50) or the host leucocyte group (median 65, range 60–80) for the duration of the experiment.

Topical administration of FCM or serum-free culture medium had no effect on the frequency of rolling, adherent or the total frequency of LAK cells or host leucocytes in the arterioles.

### *Capillaries*

There was a significant increase in the frequency of rolling LAK cells ($P < 0.05$) immediately after topical administration of TCM to the cremaster muscle (TCM vs no TCM; median 2, range 0–3 vs median 1, range 0–1; $P < 0.05$). This increase was transient and had returned to pretreatment values by the end of the experiment. There were no differences observed in the number of adherent LAK cells within the capillaries.

There was a significant increase in the frequency of both rolling (TCM vs no TCM; median 10, range 8–15 vs median 18, range 16–22; $P < 0.05$) and adherent (TCM vs no TCM; median 0, range 0–1 vs median 2, range 1–4; $P < 0.05$) host leucocytes 2 h after topical administration of TCM to the cremaster muscle. This was sustained for the remainder of the experiment.

There was no alteration in the total number of cells (flying, rolling and adherent) following administration of TCM in either the LAK cell group (median 10, range 3–20) or the host leucocyte group (median 32, range 3–40) for the remaining 2 h of the experiment.

Topical administration of FCM or serum-free culture medium had no effect on the frequency of rolling, adherent or the total frequency of LAK cells or host leucocytes in the capillaries.

### Cytotoxicity assay

Culture of splenocytes with IL-2 (LAK cells) resulted in an increased cytotoxicity towards YAC1, P815 and K1735 targets. A typical response is shown in Figure 3 in which LAK cytotoxicity against YAC1 was 51.1 ± 6.2%, against P815 was 38.3 ± 3.7% and against K1735 was 32 ± 3.2%, using an effector–target cell ratio of 50:1.

### Physiological parameters

The heart rate, blood pressure and body temperature of all animals remained constant throughout the experimental period. Mean arterial pressure was 100 ± 19 mmHg, and the mean pulse rate was 467 ± 50 beats per min. Body temperature, as measured by the oesophageal thermocouple, was within the range 36.3–37.2°C. There were no alterations in blood flow throughout the studies, as assessed qualitatively.

### DISCUSSION

The major observations of these studies were the transient and immediate increase in LAK endothelial cell interactions and the subsequent but sustained increase in host leucocyte–endothelial cell interactions in response to topical application of tumour-conditioned medium (TCM) from the murine melanoma cell line K1735 to the normal cremaster muscle microcirculation. These interactions were characterized by increased numbers of cells rolling along and adhering to the endothelium. The administration of medium from a non-tumour cell line (fibroblast-conditioned medium, FCM) had no effects on leucocyte migration or adhesion, suggesting the presence of one or more soluble tumour-specific chemoattractants for leucocytes in the tumour-conditioned medium. The increased interaction of both LAK cells and host leucocytes to the endothelium occurred in all vessel types – venules, arterioles and capillaries – although the greatest responses were observed in the venules. The effect of topical application of TCM and FCM on host leucocyte, as well as LAK cell recruitment to the cremaster muscle, was studied as host leucocytes may play an additional role in tumour rejection. It has been shown previously that the number of leucocyte–endothelium interactions appears to be dependent on the leucocyte subpopulation being observed (Sasaki et al, 1991; Fukumura et al, 1995).

Many studies have attempted to elucidate the mechanisms of lymphocyte-mediated cytotoxicity and motility in vitro (Ratner and Heppner, 1986; Goldfarb, 1989). However, few studies have attempted to investigate the migration and localization of adoptively transferred activated lymphocytes or host leucocytes into the tumour environment or surrounding normal tissue using dynamic visualization techniques, such as in vivo microscopy (Sasaki et al, 1991; Fukumura et al, 1995; Melder et al, 1995). Thus, the mechanisms by which LAK cells and host immune cells are attracted to the tumour and then promote tumour necrosis remain unclear;

© Cancer Research Campaign 1997

however, several possibilities exist. They may interact directly with and lyse the tumour cells. They may induce cytokine release, initiating tumour and endothelial cell damage, resulting in shutdown of the tumour microcirculation; this may promote the extravasation of other immune effector cells and/or result in hypoxic regions within the tumour. It is not known how many effector cells are required to either directly or indirectly lyse tumour cells, but if this leucocyte-mediated tumour cell killing requires the direct contact of effector cells with the tumour cells then the ratio may be important. However, if effector cells damage the endothelium then large numbers of tumour cells may be destroyed indirectly and the tumour–effector cell ratio may be less important. The cytotoxicity assay results demonstrate the ability of the LAK cells to induce a non-specific cytotoxicity to the three tumour cell lines in vitro, the most sensitive being YAC1.

LAK cells may also play an important role in promoting host cytotoxic T-lymphocyte (CTL) responses to tumour antigens, and recent evidence confirms that a variety of human malignancies express tumour antigens capable of stimulating antigen-specific MHC-restricted cytotoxic T lymphocytes (Brasseur et al, 1992; van der Bruggen et al, 1991). Thus, indirect cytotoxic effects may occur through LAK cells secreting a number of agents, including growth-inhibitory cytokines and chemokines which recruit or activate host cytotoxic effector cells (Hiserodt and Chambers, 1988; Mclntyre et al, 1992).

The results from this study demonstrate a transient increase in contact of the LAK cells with the endothelium, immediately after topical administration of TCM to the normal cremaster muscle. These LAK cell–endothelium interactions lasted about 15–20 min, but there was no response during this time by the host leucocytes. The increased LAK interactions with the vascular endothelium were immediate and short-lived; however, if medium was topically applied for a second time, an increase in adhesion was again observed. However, 90–120 min after TCM administration to the normal cremaster muscle, there was a dramatic increase in the numbers of host leucocytes interacting with the endothelium, i.e. rolling and adhering. The observed time difference in increased endothelial interactions between the two effector cell subtypes suggest that the responses are mediated via different mechanisms. However, both responses may be due to the presence of cytokines or chemokines in the tumour-conditioned medium (e.g. tumour necrosis factor alpha, TNF-α) stimulating either recruitment and/or activation of LAK cells and host leucocytes, as has been previously discussed. Cytokines/chemokines may also induce up-regulation of the adhesion molecules involved in leucocyte–endothelium interactions.

The recruitment of leucocytes is thought to be a multistep process. There is initial contact or tethering to the endothelium and then rolling, followed by firm adhesion and transmigration. Most rolling host leucocytes are probably granulocytes (Fiebig et al, 1991), but it is difficult to distinguish the different classes of mononuclear cells using in vivo microscopy. Leucocyte rolling is regulated by a class of adhesion molecules known as selectins (Bevilacqua and Nelson, 1993). L-selectin is expressed on monocytes, granulocytes and most lymphocytes, E-selectin is induced on cytokine-treated endothelial cells and P-selectin is expressed on stimulated and cytokine-treated platelets and endothelial cells (Springer, 1995). In vitro studies have demonstrated that vascular cell adhesion molecule-1 (VCAM-1), P-, E- and L-selectin are all involved in leucocyte rolling, but P- and E-selectin appear to be the most important in lymphocyte rolling (Springer, 1995). However, in vivo studies have demonstrated a role for P- and L-selectin but not E-selectin in mouse leucocyte rolling, during trauma and cytokine stimulation (Ley et al, 1995). Furthermore, tumours are known to produce cytokines such as interleukin 1 (IL-1) and TNF-α. TNF-α has been shown in previous studies to facilitate the binding of leucocytes to both normal and tumour endothelium (Fukumura et al, 1995; Ley et al, 1995). This is due to the up-regulation of adhesion molecules, such as E-selectin, P-selectin, ICAM-1 and VCAM-1, on both leucocytes and endothelium (Springer, 1995). Therefore, it is possible that the presence of TNF-α in the TCM would induce increased expression of both the integrins and selectins involved in leucocyte endothelial binding.

Although the dorsal skin and cranial chamber models have been used to study both normal and tumour vasculature as well as leucocyte–endothelial interactions (Wu et al, 1994; Fukumura et al, 1995), it is difficult to determine the mechanism of the response. The tumour environment is already releasing cytokines and chemokines, and so exogenous addition of these to the chamber (which has proved to be technically difficult; Fukumura et al, 1995) may not induce further interactions. In addition, it is only possible to observe the surface vessels of the tumour in vivo. The mouse cremaster muscle preparation is a modification of the original rat preparation (Baez, 1973; Meininger et al, 1987), which has been extensively used in the field of microcirculatory research. The mouse preparation can be prepared for in vivo microscopy (IVM) with an intact neurovascular supply; it is a thin preparation (50-μm) with one layer of vessels, it has a superior resolution for IVM and host leucocytes can be observed without fluorescent labelling. Previous studies have used the rat cremaster muscle microcirculation to demonstrate increased macromolecular leakage of fluorescently labelled albumin in response to human malignant ascites (Heuser et al, 1988; White et al, 1988). Thus, changes in vascular permeability, which are important in tumour biology, can also be measured using this model.

In summary, tumour-conditioned medium topically administered to the mouse cremaster muscle induces increased adhesion of both adoptively transferred LAK cells and host leucocytes. This is a tumour-specific response as fibroblast-conditioned medium and culture medium did not induce any changes in leucocyte behaviour. The present study also demonstrates that the mouse cremaster muscle microcirculation is a useful model for investigating leucocyte–endothelium interactions in response to those cytokines and chemokines that are important in tumour biology. Characterization of lymphocyte trafficking within the microcirculation and subsequent lymphocyte–endothelial cell interaction is a prerequisite for understanding the mechanism of, and the low response rate following, adoptive immunotherapy and may aid the design of more successful treatment strategies to optimize the efficacy of this treatment modality.

### REFERENCES

Baez J (1973) An open cremaster preparation for the study of blood vessels by *in-vivo* microscopy. *Microvas Res* **5**: 384–395

Basse PH, Hokland P and Hokland ME (1990) Comparison between $^{125}$IudR and $^{51}$Cr as cell labels investigations of tumour cell migration. *Nucl Med Biol* **17**: 781–791

Basse P, Herberman RB, Hokland M and Goldfarb RH (1992) Tissue distribution of adoptively transferred adherent lymphokine-activated killer cells assessed by different cell labels. *Cancer Immunol Immunother* **34**: 221–227

Bevilacqua MP and Nelson RM (1993) Selectins. *J Clin Invest* **91**: 379–387

© Cancer Research Campaign 1997

Brasseur F, Marchand M, Vanwijck R, Herin M, Lethe B, Chomez P and Boon T (1992) Human gene MAGE-1 which codes for a tumour-rejection antigen, is expressed by some breast tumours. *Int J Cancer* **52**: 839–841

Brown NJ, Ali S, Rees RC and Reed MWR (1994*a*) The distribution of unactivated lymphocytes in tumour and normal microcirculation *in vivo*. *Int J Micro: Clin Exp* **14**: 31

Brown NJ, Pollock KJ, Bayjoo P and Reed MWR (1994*b*) The effect of cryotherapy on the cremaster muscle microcirculation *in vivo*. *Br J Cancer* **69**: 706–710

Ettinghausen SE and Rosenberg SA (1986) Immunotherapy of murine sarcomas using lymphokine activated killer cells: optimisation of the schedule and route of administration of recombinant interleukin-2. *Cancer Res* **46**: 2784–2789

Ettinghausen SE, Lipford EH, Mule JJ and Rosenberg SA (1985) Recombinant interleukin-2 stimulates *in vivo* proliferation of adoptively transferred lymphokine-activated killer (LAK) cells. *J Immunol* **135**: 3623–3628

Fiebig EK, Ley K and Arfors K-E (1991) Rapid leukocyte accumulation by 'spontaneous' rolling and adhesion in the exteriorised rabbit mesentery. *Int J Microcirc Clin Exp* **10**: 127–144

Fukumura D, Salehi HA, Witwer B, Tuma RF, Melder RJ and Jain RK (1995) Tumor necrosis factor induced leucocyte adhesion in normal and tumour vessels: effect of tumour type, transplantation site and host strain. *Cancer Res* **55**: 4824–4829

Goldfarb RH (1989) Cell-mediated cytotoxic reactions. *Human Pathol* **17**: 138–145

Hayat K, Rodgers S, Bruce L, Rees RC, Chapman K, Reeder S, Dorren MS, Sheridan E, Sreenivasan T and Hancock BW (1991) Malignant melanoma and renal cell carcinoma: immunological and haematological effects of recombinant interleukin2. *Eur J Cancer* **27**: 1009–1014

Heuser LS, Miller FN and Gilley-Pietsch C (1988) Protein leak from normal vasculature due to human malignant ascites. *Am J Surg* **155**: 765–769

Hiserodt JC and Chambers WH (1988) Role of soluble cytotoxic factors in lymphokine killer cell (LAK)-mediated cytotoxicity. (Review). *Ann NY Acad Sci* **532**: 395–404

Lafreniere R and Rosenberg SA (1985) Successful immunotherapy of murine experimental hepatic metastases with lymphokine activated killer cells and recombinant interleukin-2. *Cancer Res* **45**: 3735–3741

Ley K, Bullard DC, Arbones ML, Bosse R, Vestweber D, Tedder TF and Beaudet AL (1995) Sequential contribution of L- and P-selectin to leucocyte rolling *in vivo*. *J Exp Med* **181**: 669–675

McIntyre CA, Chapman K, Reeder S, Doreen MS, Bruce L, Rodgers S, Hayat K, Schreenivasan T, Sheridan E, Hancock BW and Rees RC (1992) Treatment of malignant melanoma and renal cell carcinoma with recombinant human interleukin-2: analysis of cytokine levels in sera and culture supernatants. *Eur J Cancer* **28**: 58–63

Meininger GA, Fehr KL and Yates MB (1987) Anatomic and haemodynamic characteristics of the blood vessels feeding the cremaster muscle of the rat. *Microvas Res* **33**: 81–97

Melder RJ, Salehi HA and Jain RK (1995) Interaction of activated natural killer cells with normal and tumour vessels in cranial windows in mice. *Microvasc Res* **50**: 35–44

Ratner S and Heppner GH (1986) Mechanisms of lymphocyte traffic in neoplasia. *Anticancer Res* **5**: 475

Reed MWR, Weiman TJ, Shuschke DA, Tseng MT and Miller FN (1989) A comparison of the effects of photodynamic therapy on normal tumour vessels in the rat microcirculation. *Radiat Res* **119**: 542–552

Rosenberg SA and Lotze MT (1986) Cancer therapy using interleukin-2 and interleukin-2 activated lymphocytes. *Annu Rev Physiol* **4**: 681–694

Rosenberg SA, Lotze MT, Muul LM, Chang AE, Avis FP, Leitman S, Linehan WM, Robertson CN, Lee RE and Rubin JT (1987) A progress report on the treatment of 157 patients with advanced cancer using lymphokine activated killer cells and interleukin-2 or interleukin-2 alone. *New Engl J Med* **316**: 889–897

Sasaki A, Melder RJ, Whiteside TL, Herberman RB and Jain RK (1991) Preferential localisation of human adherent lymphokine-activated killer cells in tumour microcirculation. *J Natl Cancer Inst* **83**: 433–437

Schwarz RE, Vujanovic NL and Hiserodt JC (1989) Enhanced antimetastatic activity of lymphokine activated killer cells purified and expanded by their adherence to plastic. *Cancer Res* **49**: 1441–1446

Springer TA (1995) Traffic signals on endothelium for lymphocyte recirculation and leukocyte emigration. *Ann Rev Physiol* **57**: 827–872

van der Bruggen P, Traversari C, Chomez P, Lurquin C, De Plaon E, Van den Eynck B, Knuth A and Boon T (1991) A gene encoding an antigen recognised by cytolytic T lymphocytes on a human melanoma. *Science* **254**: 1643–1647

Wadhwa M, Bird C, Tinker A, Mire-Sluis A and Thorpe R (1993) Quantitative biological assays for individual cytokines. In *Cytokines, A Practical Approach*. Chapter 23, Balkwill FR (ed.), pp. 309–321. IRL Press: Oxford

White MJ, Miller FN, Heuser LS and Gilley-Pietsch C (1988) Human malignant ascites and histamine-induced protein leakage from the normal microcirculation. *Microvasc Res* **35**: 63–72

Wiltrout RH, Gorelik E, Brunda MJ, Holden HT and Herberman RB (1983) Assessment of *in vivo* natural antitumour resistance and lymphocyte migration in mice: comparison of $^{125}$I iododeoxyuridine with $^{111}$indium-oxine and $^{51}$chromium as cell labels. *Cancer Immunol Immunother* **14**: 172

Wu NZ, Ross BA, Gulledge C, Klitzman B, Dodge R and Dewhirst MW (1994) Differences in leucocyte–endothelium interactions between normal and adenocarcinoma bearing tissues in response to radiation. *Br J Cancer* **69**: 883–889

© Cancer Research Campaign 1997